# A Case of Lymphoma Simulating Primary Sternal Tumour

**Published:** 2014-04-01

**Authors:** Atalay Sahin, Fatih Meteroglu, Sevval Eren, Ayse Nur Keles

**Affiliations:** Dicle University Hospital, Thoracic Surgery, Diyarbakir, Turkey; Dicle University Hospital, Thoracic Surgery, Diyarbakir, Turkey; Medeniyet University, Medical School, Thoracic Surgery, Istanbul, Turkey; Dicle University Hospital, Pathology, Diyarbakir,Turkey

**Keywords:** Lymphoma, Sternum, Prosthesis

## Abstract

Any mass on the chest wall may not always be the primary local pathology. A case of lymphoma with an aggressive course may involve the sternum through local invasion and can mimic a chest wall tumour. A 15-year-old boy with mediastinal lymphoma presented with a sternal mass. Partial sternectomy with replacement by methyl methacrylate prosthesis was performed.

## INTRODUCTION

Majority of sternal tumours are malignant regardless of being primary or secondary. A slowly enlarging lump of the sternum may mimic as a primary pathology. A CT scan can also support it as sternal tumour spread to the adjacent tissues.[1,2] We managed a patient with a suspected primary sternal tumour; it was later found to be an extension of mediastinal lymphoma.

## CASE REPORT

A 15-year-old boy was referred to thoracic surgery clinic with a slowly growing non-tender mass on the anterior chest wall for 2 months. It was initially considered as a subcutaneous abscess because of slow growth and fluctuation on palpation. Ultrasound of the lesion reported as subcutaneous abscess. Needle aspiration of the lesion did not reveal any pus thus infection was ruled out. At the one-month follow-up, the overlying skin of the mass became whitish and its periphery turned reddish and inflamed. Patient also developed pain and tenderness locally. The size of the mass increased from 2x2 cm to 5x5 cm and involved the middle part of the sternum.

Laboratory tests were within the normal range. CT scan revealed a mass of 8x5 cm occupying the anterior mediastinum, eroding the sternum, extending subcutaneously as an expanding necrotic area. A thymic tissue of 4x2 cm and conglomerated lymph nodes without definite borders were also noted (Fig. 1). The second percutaneous needle biopsy of the lesion gave no aspirate. Pathological examination of the trucut needle biopsy could not prove malignancy. The patient was considered as having a sternal mass. For both the diagnosis and treatment, the anterior mediastinum was explored. Frozen section examination showed malignancy. Skin flaps were elevated over the tumour mass. The pectoral muscles were dissected from the chest wall laterally and superiorly. Total gross excision of the lesion was performed. The resection included the two thirds of sternal body, the anterior aspects of the third to sixth ribs bilaterally to the costochondral junctions. A discrete mediastinal mass of 8x10 cm and the attached thymus were also excised. The resultant bony defect of the anterior chest wall (about 10 cm) was covered with sandwich prosthesis with propylene mesh and methyl methacrylate. Medial compression of the chest from either side enabled the graft to be sutured without tension. The pectoral muscles were then re-approximated in the midline. Chest drain was left. The postoperative recovery was uneventful.

**Figure:**
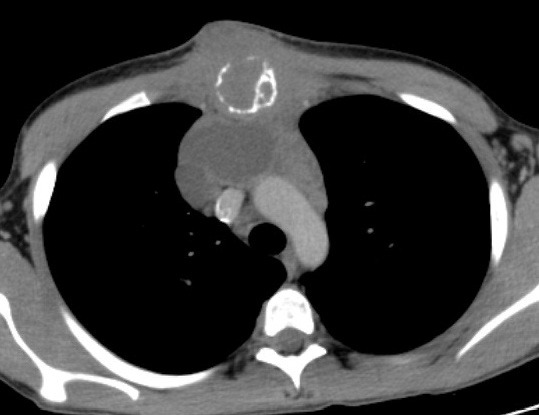
Figure 1:The mass of 8x5 cm. occupying the anterior mediastinum.

**Figure:**
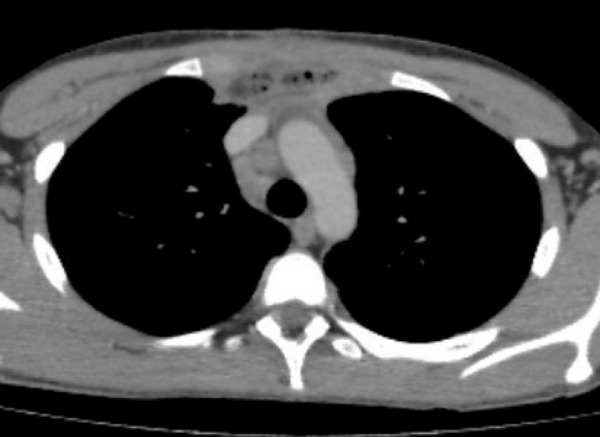
Figure 2:Postoperative CT scan.

Pathological examination of the specimen revealed primary mediastinal B-cell lymphoma invading thymus, sternum and skin. Immunohistochemically, the malignant cells were positive for CD20, Bcl-2, LCA, CD43, EMA and negative for CD3, CD10, SMA, desmin, myoglobin, suggestive of B-cell lymphoma. Following surgery and adjuvant chemotherapy, the patient was clinically without evidence of recurrent disease and had an acceptable cosmetic appearance.

## DISCUSSION

Tumours of the sternum are uncommon lesions. They present with localized pain. The masses in anterior mediastinum are more malignant compared to the other locations. These include thymoma, germ cell tumours, and lymphomas. Primary mediastinal lymphomas compose 10-20% of primary mediastinal tumours.[2-4] Primary non-Hodgkin disease in the sternum hardly occurs and believed as an extension of the disease from the anterior mediastinal lymph nodes. The cortex and sponges were not intact in our case. The disease, however, involved both the mediastinal and anterior layers of the periosteum and exhibited marked reactive membranous bone formation. The disease involved the anterior mediastinal and parasternal nodes first, and then secondarily invaded the sternum by extension through the peristernal lymphatics, which were connected to the sternal periosteum and intramedullary spaces.[5]

The index case was an example of localized lymphoma. The exploration was done to determine resectability of a primary sternal tumour. The treatment of choice for a primary sternal tumour is total sternectomy when the deeper anterior mediastinal nodes are uninvolved. Even some localized lymphomas are amenable to aggressive surgical therapy.

To conclude, anterior mediastinal lymphomas may mimic as primary sternal mass. Fine needle aspiration and trucut biopsy were non-contributory to the final diagnosis. Sternectomy with complete apparent surgical resection of the tumour and adjuvant chemotherapy worked well for our patient.

## Footnotes

**Source of Support:** Nil

**Conflict of Interest:** None declared

